# Homologous Recombination Mediates Functional Recovery of Dysferlin Deficiency following AAV5 Gene Transfer

**DOI:** 10.1371/journal.pone.0039233

**Published:** 2012-06-15

**Authors:** William E. Grose, K. Reed Clark, Danielle Griffin, Vinod Malik, Kimberly M. Shontz, Chrystal L. Montgomery, Sarah Lewis, Robert H. Brown, Paul M. L. Janssen, Jerry R. Mendell, Louise R. Rodino-Klapac

**Affiliations:** 1 Department of Pediatrics, The Ohio State University, Columbus, Ohio, United States of America; 2 Department of Neurology, The Ohio State University, Columbus, Ohio, United States of America; 3 Department of Physiology and Cell Biology, The Ohio State University, Columbus, Ohio, United States of America; 4 Center for Gene Therapy, The Research Institute at Nationwide Children's Hospital, Columbus, Ohio, United States of America; 5 Department of Neurology, The University of Massachusetts Medical School, Worcester, Massachusetts, United States of America; Medical College of Georgia, United States of America

## Abstract

The dysferlinopathies comprise a group of untreatable muscle disorders including limb girdle muscular dystrophy type 2B, Miyoshi myopathy, distal anterior compartment syndrome, and rigid spine syndrome. As with other forms of muscular dystrophy, adeno-associated virus (AAV) gene transfer is a particularly auspicious treatment strategy, however the size of the DYSF cDNA (6.5 kb) negates packaging into traditional AAV serotypes known to express well in muscle (i.e. rAAV1, 2, 6, 8, 9). Potential advantages of a full cDNA versus a mini-gene include: maintaining structural-functional protein domains, evading protein misfolding, and avoiding novel epitopes that could be immunogenic. AAV5 has demonstrated unique plasticity with regards to packaging capacity and recombination of virions containing homologous regions of cDNA inserts has been implicated in the generation of full-length transcripts. Herein we show for the first time *in vivo* that homologous recombination following AAV5.DYSF gene transfer leads to the production of full length transcript and protein. Moreover, gene transfer of full-length dysferlin protein in dysferlin deficient mice resulted in expression levels sufficient to correct functional deficits in the diaphragm and importantly in skeletal muscle membrane repair. Intravascular regional gene transfer through the femoral artery produced high levels of transduction and enabled targeting of specific muscle groups affected by the dysferlinopathies setting the stage for potential translation to clinical trials. We provide proof of principle that AAV5 mediated delivery of dysferlin is a highly promising strategy for treatment of dysferlinopathies and has far-reaching implications for the therapeutic delivery of other large genes.

## Introduction

Mutations in the dysferlin gene cause allelic autosomal recessive disorders including limb girdle muscular dystrophy type 2B (LGMD2B), Miyoshi myopathy [1], [2] and distal anterior compartment myopathy [3], [4], [5], collectively known as the dysferlinopathies. A less common phenotype of dysferlin deficiency presents with rigid spine syndrome [1], [2], [3], [4], [6]. Typically patients present in their early twenties with slowly progressive weakness and high serum creatine kinase (CK) [7]. Approximately one-third of patients become wheelchair-dependent within 15 years of onset. Clinically the heart is only mildly affected in one third of cases [8] and cognitive function is spared. The phenotypic variants with a relatively restricted distribution of muscle weakness set the stage for potential regional vascular gene replacement therapy that could impact quality of life for this disorder [9], [10]. Single nucleotide changes [11], [12], the typical DYSF gene mutation, also favors success in gene transfer serving to protect the transgene product from immunorejection.

The dysferlin gene is large, with 55 exons so far identified spanning at least 150 kb of genomic DNA. These exons predict a cDNA of approximately 6.5 kb and a protein of 2,088 amino acids [1], [2], [11], [13]. Dysferlin is a 237 kDa protein composed of a C-terminal hydrophobic transmembrane domain and a longer cytoplasmic oriented hydrophilic region with multiple C2 domains with implications for calcium and phospholipid binding [14]. Recent work has shown that loss of dysferlin compromises Ca^2+^-dependent membrane repair in skeletal muscle [15], [16]. Dysferlin-null muscle fibers fail to exclude dye entry even in the presence of Ca^2+^ strongly suggesting that Ca^2+^-dependent membrane repair requires dysferlin [17]. There is also evidence from LGMD2B patients that ultrastructural membrane defects are a present and contributing factor to disease pathology [18], [19]. The importance of this system is emphasized when considering that skeletal muscle is mechanically active and predisposed to injury; thus, a robust membrane resealing mechanism must be present. Absent or mutant dysferlin leads to impaired membrane repair and a cascade of events starting with muscle fiber necrosis resulting in muscle fiber loss and progressive limb weakness [16], [17].

Packaging limitations of AAV, estimated at ∼5 kb, present obstacles for gene replacement strategies requiring cDNA cassettes exceeding this size constraint [20], [21], [22]. Alternative methods to bypass packaging limits include miniaturizing genes and trans-splicing approaches. These tactics have often compromised function and often result in reduced levels of gene expression at standard dosing levels [23], [24], [25], [26]. A recent report suggests that the dysferlin gene can be delivered to muscle using a dual trans-splicing vector strategy with functional improvement of the defect in membrane repair [27]. A second study using AAV to deliver a naturally occurring minidysferlin protein also showed some improvement in the membrane repair defect [28]. A sentinel report indicating that AAV5 can package large transcripts up to 8.9 kb in size encouraged translational investigators to pursue gene replacement requiring inserts as large as the dysferlin gene [29], [30]. Subsequent studies showed the mechanism for AAV5 mediated delivery of genes >5 kb was homologous recombination of 5′ and 3′ products of partially packaged virions rather than ability to package intact full size genomes [31], [32]. In our own studies, we attempted to take advantage of the potential for AAV5 to deliver a dysferlin expression cassette of 7.7 kb in a single vector, including an optimized cDNA and muscle specific promoter to avoid off target effects. Our findings demonstrate highly favorable results with full restoration of dysferlin without compromise in function. In the diaphragm muscle of a mouse model of dysferlin deficiency, tetanic force was restored to normal and there was full resistance to fatigue. Importantly, the newly restored dysferlin fully repaired membrane defects in dysferlin deficient mice. In addition, rAAV5.DYSF was successfully delivered through isolated limb perfusion to the limb muscles preferentially affected in entities such as Miyoshi myopathy and distal anterior compartment myopathy. Even more proximal sites can potentially be transduced by vascular delivery as we have demonstrated in the non-human primate [10]. Of particular note, consistent with recent observations, the full-length dysferlin product that restored these favorable results was mediated by recombination of homologous region of ∼1 kb present in 5′ and 3′ packaged vector genomes, [29], [31], [32]. Herein we present our findings that AAV5 delivery is a viable treatment modality for dysferlin gene replacement with far reaching implications for other monogenic disorders caused by mutations in large genes.

## Results

### rAAV5.dysferlin gene transfer vector

**Figure 1:**
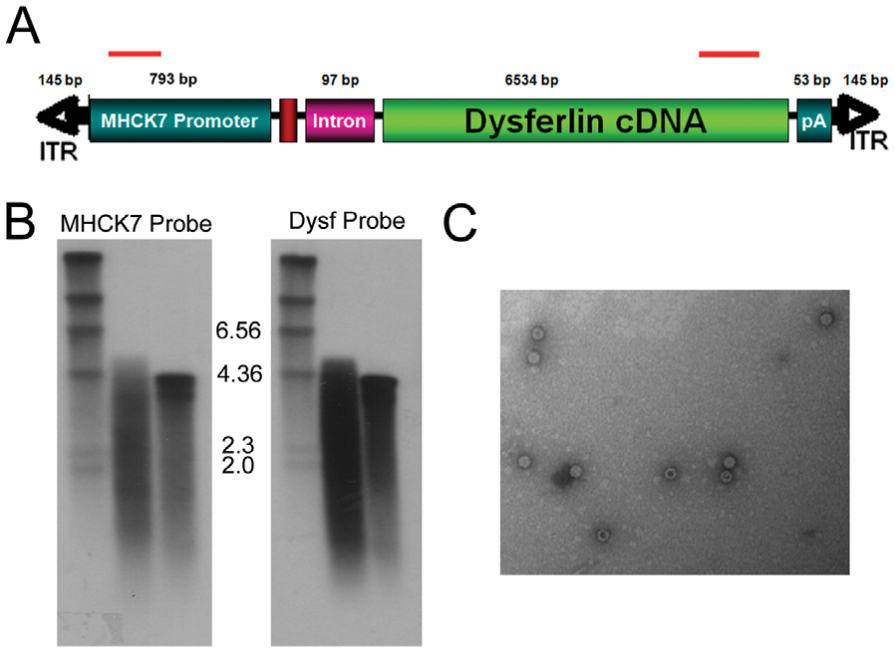
Analysis of genomes isolated from rAAV5.DYSF. DNA was isolated from rAAV5.DYSF vector preparation and used for Southern blot and PCR analysis. (A) Schematic of rAAV5.DYSF cassette. Strand specific hybridization probes used for Southern blot analysis are indicated by red bars. (B) Southern blot analysis of rAAV5.DYSF genomic DNA with 5′ MHCK7 probe (lane D, left side) and 3′ dysferlin probe (Lane D, right side). A 4.2 kb control vector genome was used as a standard for packaging (C in each blot). “M” denotes marker lane. (C) Electron microscopy of rAAV5 vector prep revealed virions with normal morphology.

**Figure 2:**
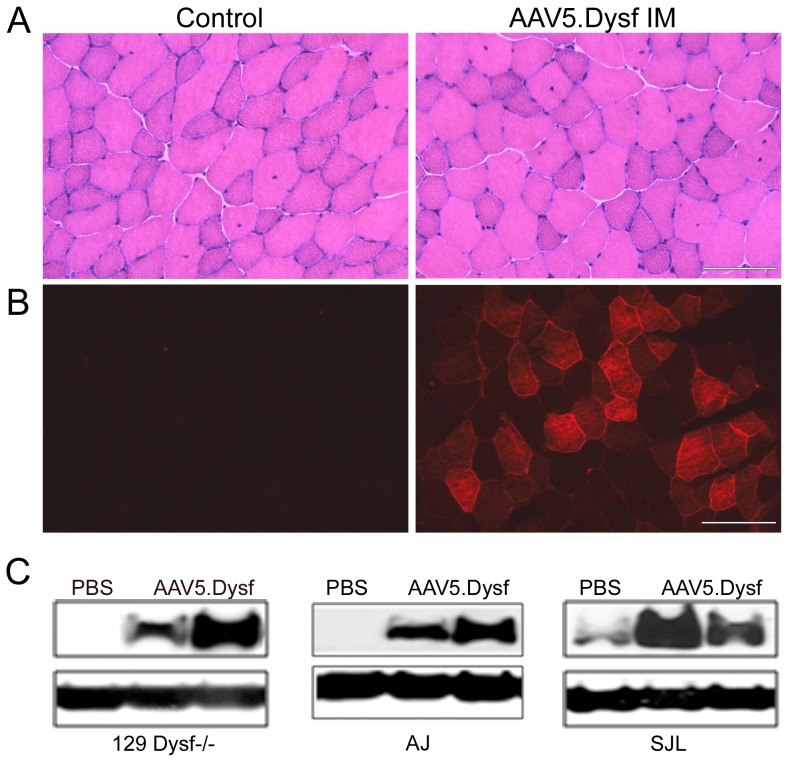
Intramuscular delivery of rAAV5.DYSF. 4–6 week old dysferlin deficient mice were injected into the tibialis anterior muscle with 10^11^ vg of rAAV5.DYSF (n = 4 per group). Endpoint analysis occurred at 4 weeks post gene transfer and analyzed by histology, immunofluorescence and western blot analysis. (A) Hematoxylin and eosin staining demonstrates very mild pathology in Dysf^−/−^ control mice which was not exacerbated by rAAV5.Dysf delivery. (B) Animals treated with rAAV5.DYSF demonstrated positive dysferlin expression by immunostaining compared to controls. (C) Western blot analysis confirmed a 237 kd full-length dysferlin band which was absent in control tissue except in SJL/J controls which have ∼15% residual dysferlin protein (PBS). Scale bar = 100 µm.

We constructed a human dysferlin cassette driven by the muscle specific MHCK7 promoter [33] (Fig. 1A). A chimeric intron was added to augment RNA processing. The cassette (7.7 kb total) was packaged into an AAV2/5 vector using standard triple transfection and purified using iodixanol gradients and ion exchange chromatography. To test whether the full-length transgene was packaged or if homologous recombination was occurring, we performed alkaline electrophoresis and southern blot analysis on vector genomes purified from the vector preparation as previously described [32]. Two probes were designed, one within the 5′ MHCK7 promoter and one located in the 3′ end of dysferlin. The findings unequivocally demonstrated that packaging did not exceed ∼5.2 kb with either probe consistent with recent reports [29], [31], [32] (Fig. 1B). AAV is known to package single strand genomes with 3′ to 5′ polarity into the pre-formed particle in an ATP dependent process starting at the 3′ ITR terminus. This is consistent with a vector “breakpoint” within this region based on the Southern blot data demonstrating packaging of up to ∼5.2 kb in length starting at a 3′ ITR termini. Despite these packaging constraints, full-length dysferlin protein was readily demonstrated in skeletal muscle consistent with a process of homologous recombination occurring within transduced myocytes to generate the full-length intact dysferlin gene (Fig. 2C) [29], [31], [32]. Using electron microscopy, AAV5 virions had normal morphology, providing further evidence that the genomes packaged did not exceed capacity (Fig. 1C).

### Intramuscular rAAV5.DYSF gene transfer to limb muscle

To test whether our AAV5.dysferlin vector could efficiently transduce muscle and express full-length intact protein, we performed intramuscular injections into the tibialis anterior (TA) muscle of 4–6 week old dysferlin deficient mice 129-Dysf^tm1Kcam/J^ (hereafter referred to as the 129Dysf^−/−^) [17], [34], [35] with10^11^ vg of rAAV5.DYSF. Animals were sacrificed after 4 weeks and muscle sections were immunostained with anti-human dysferlin antibody and assessed for histopathological changes. As shown by previous studies, Dysf^−/−^ mice demonstrate very mild pathology in young mice which is limited to a small number of centrally nucleated fibers and isolated necrotic fibers [15], [35]. There was no evidence of toxicity in the muscle following AAV5.Dysf gene transfer (Fig. 2A). In control muscles, 3.8±0.8% of fibers had central nuclei versus 2.9±1.6% in treated muscles and no evidence of necrosis. Widespread dysferlin expression was achieved which correctly localized to the sarcolemma, whereas there was no dysferlin expression in contralateral control muscles (Fig. 2B). Cytoplasm dysferlin was also encountered as noted in newly regenerating fibers in dystrophic patients [36] and is consistent with other studies demonstrating exogenously expressed dysferlin [27], [28]. The number of muscle fibers transduced was quantified with 67.3±14.4% expressing dysferlin. Western blot analysis confirmed the immunostaining results. Treated mice had a well-defined 237 kDa band that was absent in PBS control-treated animals (Fig. 2C). Efficient gene transfer was confirmed in two other models of dysferlin deficiency, SJL-Dysf and A/J for potential use in pre-clinical outcomes analyses (Fig. 2C). As expected, SJL-Dysf mice express residual dysferlin protein which increased following gene transfer (Fig. 2C).

**Figure 3:**
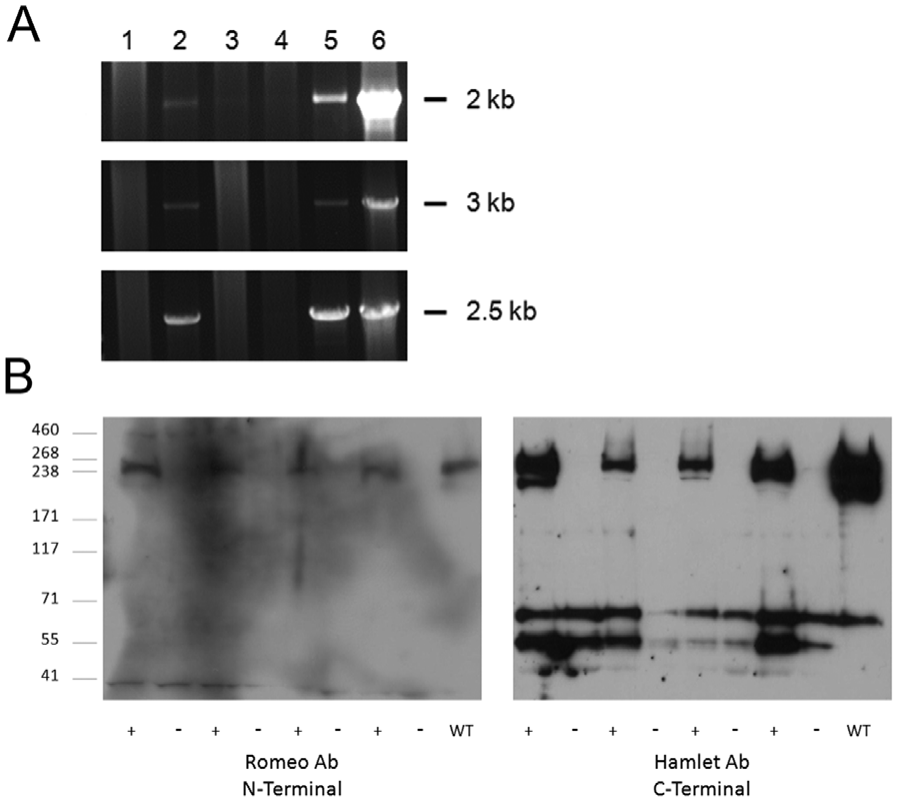
Lack of truncated Dysferlin mRNA or protein in injected muscle. (A) RNA was extracted from injected tissue, converted to cDNA, and analyzed by PCR using 3 overlapping primer sets (Top to Bottom, 5′ to 3′ end) which showed that the entire Dysferlin transcript was amplified in injected muscle. Lane 1 – no template, lane 2 – injected muscle, lane 3 – injected muscle with no reverse transcriptase enzyme, lane 4 – uninjected muscle, lane 5 – human muscle control RNA, lane 6 – pAAV.MHCK7.DYSF control DNA. (B) Protein was extracted from injected tissue and analyzed by western blot. 4 mice were analyzed following injection with rAAV5.Dysf. A wild-type mouse muscle sample was used as a control. Both an N-terminal antibody and a C-terminal antibody were used for protein analysis in these muscles. Only the full-length Dysferlin band is unique to injected muscles.

To fully address whether the recombination events that are leading to dysferlin expression are specific in generating only full-length transcript and protein, we performed RT-PCR and western blot analysis on samples isolated from injected tissue. We extracted RNA from treated and control injected TA muscles and following cDNA conversion, three overlapping PCR products specific for human dysferlin were amplified in AAV5 injected tissue and not in control tissue (Fig. 3A). The products were sequenced and found to be 100% identical to the human dysferlin transcript encoded by the transgene cassette (Fig. S1). Moreover, only full-length dysferlin was present on western blot using both N and C-terminal dysferlin antibodies (Fig. 3B).

### Functional outcomes of dysferlin deficiency in skeletal muscle

Development of a therapeutic transgene intended for clinical application requires preclinical efficacy with a functional outcome measure. Prior to the initiation of our study, a physiological outcome measure for tetanic force or resistance to contraction induced injury had not been defined in dysferlin deficient mice. We first assessed skeletal muscle function using the extensor digitorum longus muscle (EDL) in 6 month old animals in all three dysferlin-deficient mouse strains and corresponding strain controls (8 per group). Animals were euthanized and the EDL was dissected for *in vitro* force measurements. Dysferlin deficient muscles showed no deficits in maximum isometric force compared to strain controls when normalizing for the cross-sectional area of the muscle (ANOVA, P>0.05) (Fig. S2A). After assessment of specific force, the muscles were then subjected to mechanical damage by repetitive eccentric contractions. Dysferlin deficient muscles showed no significant reduction in force generation by repetitive eccentric contractions compared to their corresponding strain control muscles (Fig. S2B–D, 2-way analysis of variance, P>0.05).

**Figure 4:**
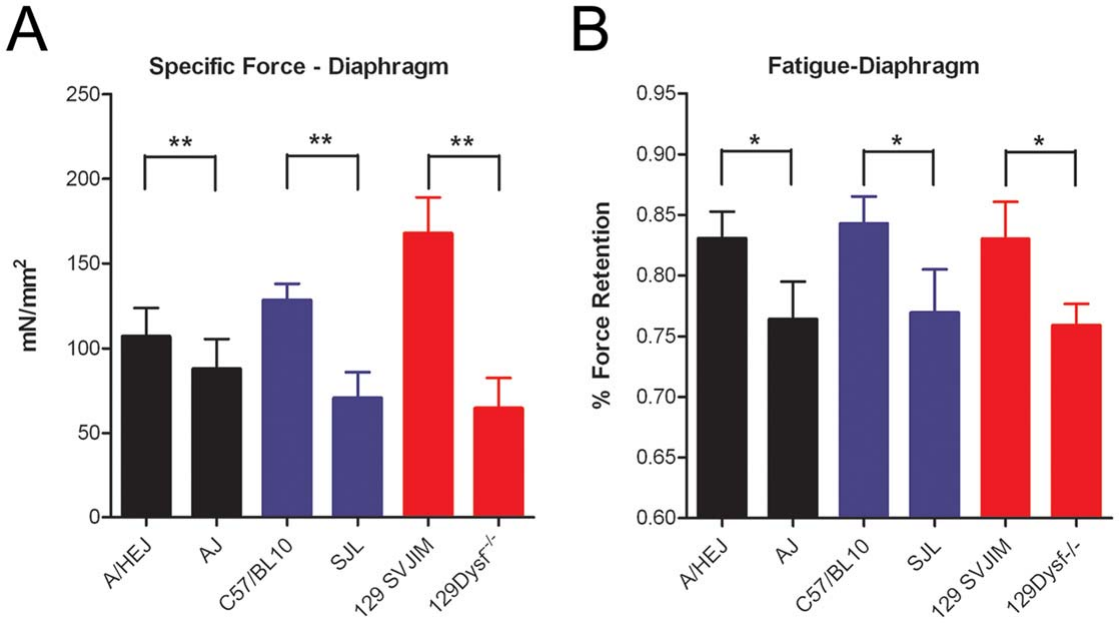
Dysferlin deficient diaphragms exhibit significant impairment in force generation and resistance to fatigue. Diaphragms from SJL-Dysf, A/J, and 129-Dysf^−/−^animals along with control animals C57/BL10, A/HeJ, and 129S1/SvImJ (8 per group) were isolated. (A) All three models demonstrated reduced specific force compared to their corresponding strain control (t-test P<0.05). (B) Fatigue induced by stimulation every second revealed significantly lower resistance to fatigue in all three Dysferlin deficient models compared to their corresponding strain controls (2-way analysis of variance, P<0.001). Force retention following ten contractions is shown.

Functional deficits were further examined in the diaphragm muscle that exhibits progressive signs of dystrophy [35]. Diaphragm strips from 24 week old 129-Dysf^−/−^, SJL-Dysf, and A/J animals along with corresponding strain control animals (8 per group) were dissected with rib attachments and central tendon intact. A 1–2 mm wide section (from rib to tendon) of diaphragm was isolated, and attached to a force transducer. The muscle was stretched to the length where twitch contractions were optimal, allowed to rest for 10 minutes, and subjected to a protocol consisting of a series of eight tetanic contractions occurring at 2 minute intervals, each with duration of 500 ms. Following a 5 minute rest, the muscle underwent a fatigue protocol which measured the force exerted by the muscle when stimulated every second for 90 seconds (500 ms tetanus at 100 Hz). All measurements were normalized to cross sectional area. Dysferlin deficient diaphragms demonstrated significant deficits in maximum isometric force compared to strain controls (ANOVA, P<0.05) (Fig. 4A). Dysferlin deficient diaphragms were also significantly more affected (larger loss of force) by muscle fatigue compared to their corresponding strain control muscles (Fig. 4B, 2-way analysis of variance, P<0.001).

**Figure 5:**
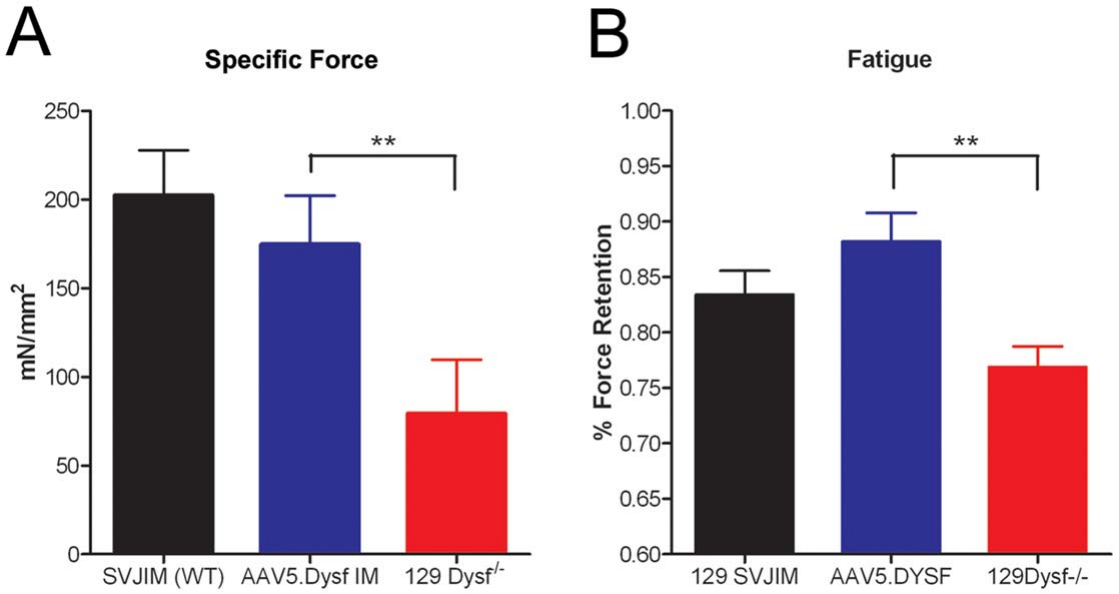
rAAV5.DYSF delivered directly to Dysf^−/−^ diaphragm corrects tetanic force and resistance to fatigue. The diaphragm of 10 week old dysferlin deficient mice (129-Dysf^−/−^) (n = 6 per group) was treated with 10^11^ vg of rAAV5.DYSF via a peritoneal incision. Ten weeks post gene transfer, diaphragm muscle strips were harvested and subjected to a protocol to assess tetanic force and resistance to fatigue. (A) rAAV5.DYSF treated diaphragms demonstrated significant improvement in tetanic force (P>0.05, ANOVA) which was not different from wild-type force (129S1/SvImJ). (B) rAAV5.DYSF treated diaphragms demonstrated significant resistance to fatigue compared to untreated Dysf^−/−^ controls (2-way analysis of variance, P<0.001) and were not different than SVJIM wild-type controls. Force retention following ten contractions is shown.

Functional deficiency in the diaphragm provided a substrate to test AAV5.DYSF gene transfer in dysferlin deficient mice. To deliver the vector to the diaphragm, a single incision was made from the base of the sternum to just above the pelvis in 10 week old 129-Dysf^−/−^ mice (n = 6 per group). The diaphragm was identified and 30 µl of the vector preparation (2×10^11^ vg) was delivered using a 32 gauge needle prior to closing the abdominal wall. Animals were sacrificed 10 weeks post treatment and the diaphragm was isolated and subjected to maximum tetanic contractions and a muscle fatigue protocol. Treated diaphragms demonstrated a significant improvement in tetanic force (Fig. 5A, P<0.05, ANOVA) which was not different from WT force (129S1/SvImJ). Treated diaphragms also demonstrated a significant improvement in resistance to fatigue compared to saline treated controls (Fig. 5B, 2-way analysis of variance, P<0.001), and were not significantly different than WT strain controls (129S1/SvImJ).

### Restoration of membrane repair following intramuscular delivery of AAV5.DYSF

**Figure 6:**
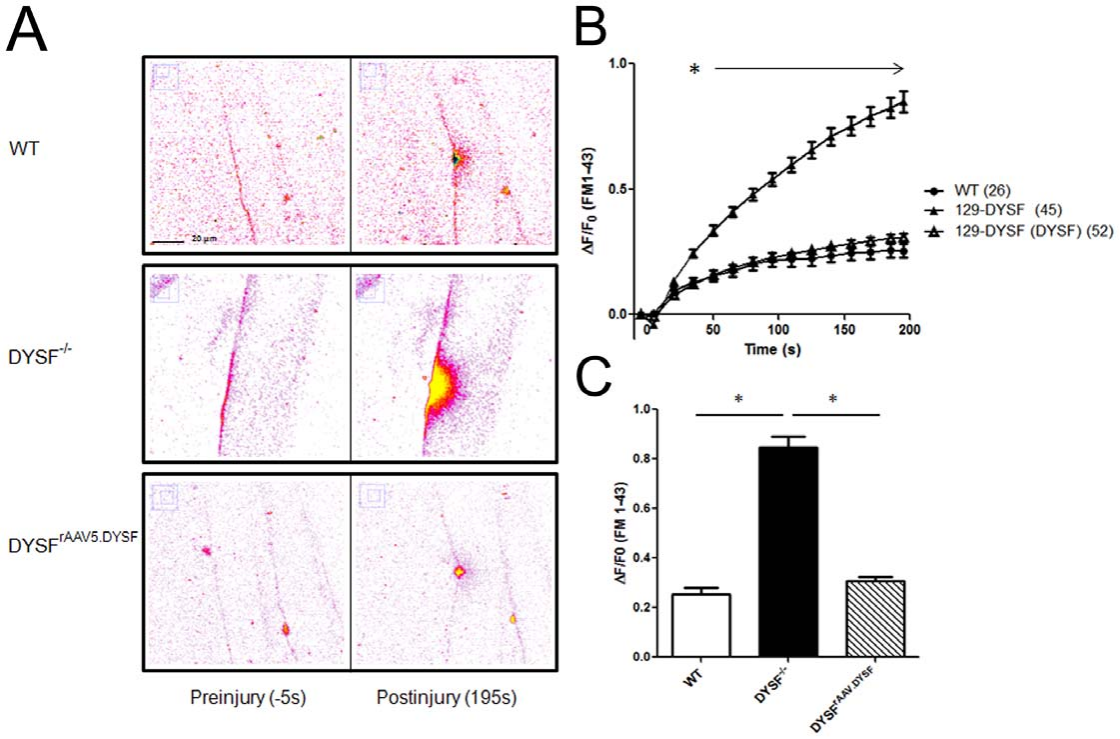
Recovery of membrane repair following rAAV5.DYSF injection in muscle. (A) Individual flexor digitorum brevis fibers were isolated from WT, 129-DYSF^−/−^, and 129-DYSF^rAAV.DYSF^ mice and the sarcolemma was damaged in the context of a solution containing FM 1–43. Images were taken before injury (−5 s) and every 5 s for a total of 195 s. Representative initial and final images for WT, 129-DYSF^−/−^, and 129-DYSF^rAAV.DYSF^ fibers are shown. (B) Fluorescence intensity from injured fibers was measured using ImageJ software, converted to change in fluorescence intensity over time, and then graphed. For clarity, only values corresponding to 15 s intervals are shown following injury. (C) The total change in fluorescence intensity over time is shown for WT, 129-DYSF^−/−^, and 129-DYSF^rAAV.DYSF^ fibers at 195 s post-injury. (2-way analysis of variance, P<0.05).

We next evaluated the ability of AAV5.DYSF treatment to restore membrane repair capability in dysferlin deficient muscle. To test this we performed a membrane wounding/resealing assay using a multi-photon laser scanning microscope on fibers isolated from the flexor digitorum brevis (FDB) muscle. We injected 2 month old 129-Dysf^−/−^ (5 per group) with 3×10^10^ vg AAV5.DYSF in the (FDB) muscle. WT (129S1/SvImJ), 129-Dysf^−/−^ and AAV5.DYSF treated 129-Dysf^−/−^ mice were sacrificed 8 weeks post-treatment, the FDB muscle was isolated, and individual fibers were isolated following collagenase treatment. Sarcolemmal damage was induced in isolated fibers using the multiphoton laser (20% power for 5s) in the presence of FM 1–43 dye. A small area of fluorescence was detected in all fibers immediately after laser injury at the site of damage. In WT muscle fibers the sarcolemma is repaired and the amount of dye that integrates into the membrane stabilizes (Fig. 6). In contrast, dye continued to integrate into the sarcolemma of the fibers from 129-DYSF^−/−^ muscle which resulted in significantly higher levels of fluorescence following a 3 min time course (Fig. 6). Expression of dysferlin from AAV5.DYSF-transduced fibers resulted in membrane repair that was equivalent to WT fibers further indicating that the exogenously expressed protein is fully functional (Fig. 6). Taken together, these data demonstrate that a large, potentially therapeutic cDNA can be delivered to muscle and efficiently express full-length functional dysferlin protein in muscle using AAV5.

### Regional vascular delivery of rAAV5.DYSF

**Figure 7:**
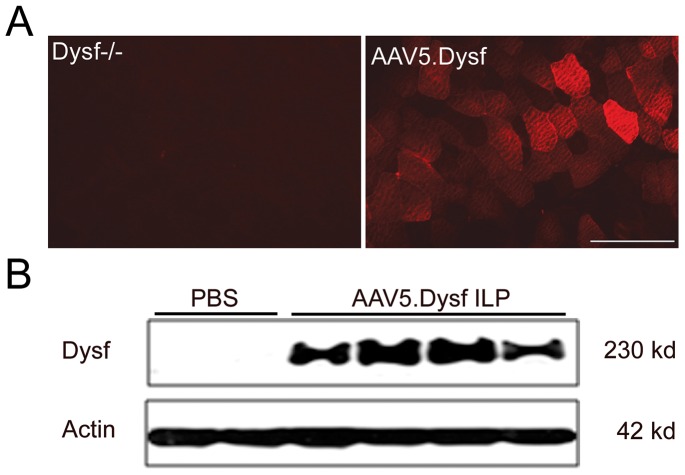
Vascular delivery of rAAV5.DYSF effectively transduces the lower hindlimb muscles of Dysferlin deficient mice. rAAV5.DYSF (10^12^ vg) was delivered via the femoral artery to the hindlimb of 3–4 week old Dysf^−/−^ mice. (A) Four weeks post transfer, immunostaining demonstrated dysferlin expression in treated animals (right). (B) Western blot confirmed 237 kd dysferlin protein in treated muscle which is absent in PBS controls. Scale bar = 100 µm.

Translational goals of dysferlin gene replacement require vascular delivery to reach multiple muscle groups. Therefore, we addressed whether rAAV5.DYSF could effectively cross the vascular barrier and transduce the lower hindlimb muscles of dysferlin deficient mice. rAAV5.DYSF (10^12^ vg) was delivered via the femoral artery to the hindlimb of Dysf^−/−^ mice using a methodology previously described for other AAV serotypes and transgenes [9]. Four weeks post transfer; animals were analyzed for dysferlin expression. Efficient transduction of the tibialis anterior muscle was observed (Fig. 7A) with 75.0±16.2% muscle fibers expressing dysferlin. Western blot analysis again confirmed protein expression as evidenced by a clear 237 kDa band corresponding to full-length dysferlin (Fig. 7B).

## Discussion

AAV-mediated gene delivery remains a potential treatment option for some patients with muscular dystrophy. Challenges, however, for DYSF gene replacement relate to AAV packaging limits for genomes significantly larger than wild-type length (4.8 kb). Fortunately, the sentinel report of Alloca *et al.* brought enhanced packaging by AAV5 to light and gave new hope for expanding the capacity of AAV gene transfer for monogenic disorders of large genes with the potential to be carried to the bedside [30]. In their studies, efficient packaging, transduction, and expression of an 8.9 kb cassette of a large inherited gene causing blindness implied the ability to overcome the hurdle of a 5 kb size limitation for AAV for replacement therapy. Our current study along with three others [29], [31], [32] have demonstrated that homologous recombination of partially packaged genomes is the mechanism responsible for the generation of full-length transcripts rather than oversized packaging of the whole genome. Regardless, AAV5 does exhibit enhanced plasticity regarding packaging constraints which is likely contributing to its ability to mediate production of full-length transcripts in muscle following homologous recombination [30]. With regard to neuromuscular diseases, the findings provided a new perspective for conditions caused by mutations of large genes. DMD is the most common severe childhood muscular dystrophy and would seem to benefit from expression of the larger transcripts than mini- and micro-dystrophins that only partially restore physiologic function in the mdx mouse [9], [23]. Less common disorders, such as titin deficiency causing LGMD2J, and variants of congenital muscular dystrophy such as phenotypes caused by LARGE gene mutations would also benefit from expression of the full length protein [37], [38], [39]. Because of the success of Alloca *et al.*, it was our intent to take advantage of the transduction capabilities of AAV5 for skeletal muscle [30]. We targeted the dysferlin gene because of its relative frequency amongst the LGMDs [5], [40]. Our findings are supportive of the unique properties of AAV5 to express a 6.5 kb cDNA producing full length protein but the mechanism for expression of the dysferlin gene pointed in a different direction than reported for the series of retinal genes protecting against blindness [30]. Our studies confirmed that AAV5 followed traditional packaging limits for DYSF because analysis of packaged virions was consistent with a packaging limitation of ∼5 kb. Thus, AAV5 virions transducing muscle fibers contained partial dysferlin vector genomes that mapped to both the 5′ and 3′ ends of the expression cassette and produced an intact expression cassette likely through homologous recombination upon reaching the nucleus [29], [31], [32]. This enabled translation of the full length 237 kDa protein demonstrated by western blots following gene transfer. The findings were equally robust following intramuscular and intravascular delivery, and the validity of the newly expressed protein was confirmed by its full restoration of physiologic function in the diaphragm and membrane repair capabilities in skeletal muscle.

Of particular importance, the potential for DYSF gene replacement has also been demonstrated by Lostal *et al.* with their comparable findings demonstrating expression of full-length dysferlin [27]. These researchers used a dual vector strategy permitting the dysferlin cDNA to be split at the exon 28/29 junction and cloned it into two independent AAV vectors carrying the appropriate splicing sequences. Their work tested the efficiency of dual packaging into AAV2/1 by intramuscular injection of both vectors into a dysferlin-deficient mouse and a novel strategy using systemic delivery of dual vector administration of both rAAV2/1 and rAAV2/9. Overall, both IM and systemic vascular delivery via the tail vein of dysferlin-deficient mice showed improvement of muscle pathology. Their study also noted an improvement in membrane repair in the FDB that did not reach WT control levels. By systemic delivery they found low levels of transduction (1–4%) which is a current limitation of the dual vector approach. In a clinical setting, higher levels of gene expression may be required to achieve a clinically meaningful outcome. Lostal *et al* suggested that 30% levels of dysferlin may be needed (26).

An additional report by Krahn *et al.* described a human minidysferlin protein that was identified in a patient with late-onset moderate dysferlinopathy [28]. The 73 kDa protein lacks the consensus dysferlin N-terminus but maintains the wild-type C-terminus including the last two C2 domains and transmembrane domain. The authors packaged this minidysferlin cDNA in a cassette containing a C5.C12 promoter and delivered it to A/J dysferlin deficient mice using AAV2/1 and AAV2/9. Expression of the protein was confirmed in the TA and found to localize primarily to the cytoplasm of muscle fibers and to a lesser extent in the sarcolemma. An examination of function revealed that the minidysferlin led to partial improvement in the membrane repair deficits accompanying the dysferlin-null FDB muscle fibers [28].

Our studies demonstrate clinical applicability for AAV5.DYSF gene transfer for dysferlin deficiency. There are several advantages to using AAV5 to deliver the full-length dysferlin cDNA. One particular advantage is the ability to deliver the entire cassette with one vector, significantly reducing viral load compared to the dual vector strategy. This is especially important with regards to translation in terms of feasibility of vector production and limiting capsid exposure for patients. A second advantage relates to full-length protein versus a miniature version. Although mini-genes are desirable for practicality of standard AAV delivery, there is a compromise in protein function [28] and the potential for novel epitopes that may be immunogenic when delivered to patients [41]. One potential concern with AAV5.dysferlin delivery is the presence of non-recombined vector genome fragments; however we showed only one mRNA and protein species was present in transduced muscle. As with any other naturally occurring truncated transcript that could be produced; elimination would occur by nonsense mediated decay. An informal discussion with the FDA (Rodino-Klapac and Mendell personal communication) defined a potential path for a dysferlin clinical gene therapy trial assuming no problems were encountered in the toxicology/biodistribution studies done with the same rigor as other approved AAV vectors [41], [42], [43].

In conclusion, we have shown that AAV5.dysferlin delivery is a very promising therapeutic approach that could restore functional deficits in dysferlinopathy patients. Specific muscle groups could be targeted by intramuscular delivery for dysferlin phenotypes that include Miyoshi myopathy and distal anterior compartment myopathy. In addition, based on our experience using fluoroscopy guided vascular delivery studies in the non-human primate we can thread the intravascular catheter to the take off point of specific vessels ^9^. This opens up clinical pathways for gene delivery to particular muscle groups relevant to either LGMD2B or Miyoshi myopathy including quadriceps and hamstring muscles, and the anterior or the posterior compartments of the lower limb.

## Materials and Methods

### Dysferlin gene construction

The full-length human dysferlin cDNA was used for all gene transfer studies. The MHCK7 promoter is derived from the MCK promoter with an additional 5′ enhancer from the myosin heavy chain (gift of S. Hauschka) [33]. The cassette includes a consensus Kozak sequence, an SV40 chimeric intron, and a synthetic polyadenylation site (53 bp). The dysferlin expression cassettes was cloned between AAV2 ITRs using flanking Xba I restriction enzyme sites in a plasmid derived from pCMVβ (Clontech). Msc I/Sma I restriction enzyme digestions were used to confirm ITR integrity.

### rAAV Vector production

rAAV2/5 vectors were produced by a modified cross-packaging approach whereby the AAV type 2 vector genome can be packaged into multiple AAV capsid serotypes [21]. Production was accomplished using a standard 3 plasmid DNA/CaPO_4_ precipitation method using HEK293 cells. Cells were maintained in DMEM supplemented with 10% fetal bovine serum (FBS) and penicillin and streptomycin. The production plasmids were: (i) pAAV.MHCK7.dysferlin, (ii) rep2-cap5 helper plasmid encoding serotype 5 capsid proteins, and (iii) an adenovirus type 5 helper plasmid (pAdhelper) expressing adenovirus E2A, E4 ORF6, and VA I/II RNA genes. Virus was purified from 1% DOC detergent lysed clarified cell pellets using iodixanol gradients and anion-exchange column chromatography as previously described [44]. A quantitative PCR-based titration method was used to determine an encapsidated vector genome (vg) titer utilizing a Prism 7500 Taqman detector system (PE Applied Biosystems) [45].

### Vector genome analysis

DNA was isolated from the rAAV5.DYSF vector preparation as previously described [32] with some modifications. Briefly, 5×10^11^ vg was subjected to DNase (Invitrogen) treatment (225 U at 37°C 30 min, 95°C 10 min, 4°C) followed by Proteinase K (Invitrogen) treatment (20 µg at 50°C 60 min, 95°C 20 min, 4°C). Vector DNA was purified using a Qiagen PCR purification kit according to the manufacturer's instructions (Qiagen Inc, Cat No. 28104). Southern blot analysis was performed as previously described [32]. Fifteen ng of probe (MHCK7 and Dysferlin) was used for each Southern blot hybridization.

### RNA analysis

RNA was isolated from TA muscles using an RNeasy® Mini Kit (QIAGEN). cDNA was synthesized from 100 ng RNA using the High Capacity cDNA Reverse Transcription kit (Applied Biosystems™) and PCR was performed using PFU Ultra II (Strategene) with the following primers: Dysf 1F (5′ATGCTGAGGGTCTTCATCCTCTA3′) – Dysf 1976R (5′ACCACAGGTTTCACGTTACC3′), Dysf 1957F (5′GGTAACGTGAAACCTGTGGT3′) – Dysf 5005R (5′TGTTCTCCAGGTCGACGAC3′), Dysf 3820F (5′TTGAGCTCATCCAGAGAGAGAAGC3′) – Dysf 6243R (5′TCAGCTGAAGGGCTTCACCAG3′). Human skeletal muscle total RNA (Biochain) and the pAAV.MHCK7.Dysf plasmid (50 ng) were used as positive controls. PCR products were gel purified using the Qiaquick Gel Extraction Kit (QIAGEN) and sequenced using the following primers: Dysf 1F, Dysf 499R (5′ CAGTGAGTCCCTGGTCCTCT3′), Dysf 501F (5′ AGATGAGGCGGAGCCATTCC3′), Dysf 900F (5′GTTCCGGATGGACGTGGGCA3′), Dysf 1976R (5′ACCACAGGTTTCACGTTACC3′), Dysf 1957F, Dysf 2400R (5′TCCCTGCAGCATCCAGATGA3′), Dysf 3339R (5′CCTCAAGGGCAAACACAGCTGC3′), Dysf 3377F (5′TTCCATGTCCGTCTCCACCTTG3′), Dysf 3820F, Dysf 4750F (5′ ATTGTCCGAGCATTTGGCCT3′), Dysf 5005R (5′TGTTCTCCAGGTCGACGAC3′), Dysf 5750F (5′ CCTTTGATGATTTTCTGGGC3′), and Dysf 6243R. The sequenced cDNA from injected tissue was then compared to the reference Dysferlin sequence (Genbank# NM_003494.3) using the ClustalW online alignment tool.

### Dysferlin deficient mouse strains

Stocks of A/J, A/HeJ, SJL/J, C57BL/10, 129S1/SvImJ, and 129-Dysf^−/−^ mice were bred and maintained as homozygous animals in standardized conditions in the Vivarium at the Research Institute at Nationwide Children's Hospital. They were maintained on Teklad Global Rodent Diet (3.8% Fiber, 18.8% Protein, 5% fat chow) with a 12∶12 h dark∶light cycle. Procedures used in the experiments were approved by the Institutional Animal Care and Use Committee at Nationwide Childrens Hospital (AR08-00017).

### rAAV5.DYSF intramuscular gene transfer

The anterior compartment (containing tibialis anterior muscle and EDL) of 4–6 week old dysferlin deficient mice was injected with 10^11^ vg of rAAV5.DYSF diluted in normal saline buffer (50 µl volume). Control mice were sham injected with normal saline. The TA and EDL muscles from both limbs were harvested at 4 weeks post administration to assess efficiency of gene transfer. For histological analysis, muscles were embedded in 7% gum tragacanth and flash frozen in isopentane cooled in liquid nitrogen. Cryostat sections (12 µm) were collected for immunohistochemistry.

### Intramuscular injections into the diaphragm

Ten week old dysferlin deficient mice (n = 6 per group) were treated with 2×10^11^ vg of rAAV5.DYSF via a peritoneal incision. Anesthetized mice (Ketamine/Xylazine 100 mg/kg and 10 mg/kg, respectively) were secured to a warm surgical table and the abdomen prepped and draped in a sterile fashion. A single incision was made from the base of the sternum to just above the pelvis (approximately 1 cm incision). The diaphragm was identified and 30 µl of the vector preparation in sterile saline was delivered using a 32 gauge needle. The abdominal wall was closed with 4.0 Vicryl Plus continuous sutures and skin wound closed with sterile surgical staples. Mice were treated with a post-op dose of Buprenorphine 0.01 mg/kg subcutaneously for pain. Ten weeks post gene transfer; diaphragm muscle strips were harvested and subjected to a protocol to assess resistance to fatigue.

### Membrane Repair Assay

The ability of the sarcolemma to repair following injury was assessed on at least 5 129-SVLMJ, 129-Dysf−/−, and 129-Dysf^rAAV5.DYSF^ mice at 4.5 months of age. Individual fibers were isolated from the flexor digitorum brevis muscle following treatment with a 2% collagenase solution. The fibers were washed in PBS and placed in glass bottom dishes in the presence of 2.5 µM FM® 1–43 (Invitrogen®) with or without 1.5 mM Ca2+. Membrane damage was induced with a FluoView® FV1000 two-photon confocal laser-scanning microscope (Olympus). A circular area (diameter, 5 µm) on the edge of the sarcolemma was irradiated at 20% power for 5 s. Images were captured 5 s prior to injury and every 5 s after injury until 3 min post irradiation. For every image, the fluorescence intensity surrounding the site of damage was analyzed (ImageJ). At least 25 fibers were evaluated for each condition (2-way analysis of variance, P<0.05).

### Vascular delivery

Eight adult dysferlin deficient mice (4–6 weeks of age) were perfused with 10^12^ vg rAAV5.DYSF in 100 µL of normal saline as described [9]. Briefly, mice were anesthetized and the femoral bundle was visualized via a small incision proximal to the mid-thigh. Blood flow through the femoral artery was controlled by catheter placement using a customized heat pulled polypropylene 10 (PE-10) catheter placed into the femoral artery. Prior to vector administration, the arterial catheter was flushed (pre-flush) with 100 µl sterile normal saline. Immediately prior to vector administration all blood flow to the extremity was impeded (isolated limb perfusion – ILP) by tightening the ligature at the mid-thigh. rAAV5.DYSF was perfused through the femoral artery in 100 µl of sterile Tris buffered saline administered at a rate of approximately 2 µl per second (over 60–80 seconds). After 10 minutes of maintained vascular occlusion, 100 µl of normal saline was administered to the arterial catheter (again at about 2 µl per second) as a post-flush and the tourniquet was then released. The wound was flushed with sterile normal saline and closed with a 6-0 proline suture.

### Immunohistochemistry

Immunostaining for dysferlin was performed on all transduced tissue to assess efficacy of gene transfer. Tissue sections were incubated with dysferlin rabbit anti-human monoclonal (Lifespan Biosciences, Inc., Cat #LS-C138735) antibody to detect dysferlin at a dilution of 1∶100 in block (20% goat serum, 0.1% triton X-100 in PBS) applied for 1 hour at 25°C, in a wet chamber. Sections were washed with PBS 3×20 minutes, reblocked, and then incubated for 30 minutes at 25°C with an Alexa 568 goat anti rabbit antibody at a dilution of 1∶300 (Molecular Probes). Sections were washed in PBS for 3×20 minutes and mounted with Vectashield mounting medium (Vector Laboratories). Images were captured with a Zeiss Axioskop2 Plus Microscope and AxioCam MRC5 camera. Four random 20× images (each field with an average of 150 muscle fibers) were captured per muscle and the number of fibers with dysferlin staining was counted and expressed as percent of total number of fibers.

### Morphometrics

Centralized nuclei counts were performed on sections of TA muscles stained with hematoxylin and eosin (H&E) from AAV5.DYSF treated Dysf^−/−^ animals. TAs from the contralateral extremity served as controls. Four random 20× fields of 12 µm sections for each muscle were captured and the number of fibers with central nuclei counted and expressed as a percentage of the total number of fibers.

### Western Blot Analysis

The TA was isolated from both the treated and contralateral limbs. From each muscle, fresh frozen serial tissue sections were taken for both immunohistochemistry and for western blot analysis. Muscle samples harvested from rAAV5.DYSF and control groups were compared with wild-type mice for levels of vector-mediated dysferlin compared to endogenous dysferlin. Protein (25 µg) extracted from treated and control samples was separated by SDS-PAGE (3–8% Novex NuPAGE gradient gels, Invitrogen), blotted on PVDF membrane and probed with dysferlin rabbit anti-human monoclonal (Lifespan Biosciences, Inc.) primary antibody at a dilution of 1∶1000, NCL-Hamlet (Novocastra) primary antibody at a dilution of 1∶1,000 or actin antibody (NCL-MSA) at a dilution of 1∶6,000 followed by horseradish-peroxidase labeled goat anti-mouse IgG or hoseradish-peroxidase labeled goat anti-rabbit IgG (1∶5,000, Millipore). Immunoreactive bands were detected with ECL Plus detection system (GE Healthcare) and signal captured on Hyperfilm ECL (Amersham).

### Force generation and protection from eccentric contractions in EDL

SJL-Dysf, A/J, and 129-Dysf^−/−^ animals along with control animals C57/BL10, A/HeJ, and 129S1/SvImJ (8 per group) were assessed for physiological deficits in the extensor digitorum longus (EDL) muscle at 24 weeks (when histopathological features are present) as previously described [9], [46]. Mice were euthanized and the EDL was removed, the tendons sutured, and bathed in oxygenated circulating Krebs-Henseleit solution at 30°C in an organ bath. For the procedure, one end of the muscle was tied to a force transducer and the other to a high-speed linear servo-controlled motor. The muscle was mounted in the set-up at slack length with a resting tension of 1 g for 10 minutes without electrical stimulation. Stimulation was delivered via two parallel platinum-iridium electrodes on either side of the muscle. Muscles were adjusted to optimum length (L0), defined as the length for maximal twitch and subjected to an isometric tetanus of 150 Hz. Following a 5 minute rest period, muscles were subjected to an eccentric contraction protocol consisting of a series of 10 isometric 700 ms tetani, at 2 minute intervals, with a 5% lengthening of the muscles (0.5 fiber length per second for duration of 200 ms) when maximal force had developed at 500 ms. After the tetanus ended (at t = 700 ms), the muscle was brought back to initial length (at the same speed as the stretch), allowing for full relaxation to the initial length. For comparative purposes, all force measurements are expressed per unit cross-sectional area (normalized isometric force or tension: mN/mm^2^). Cross-sectional area (CSA) is calculated using the following equation, CSA = (muscle mass in g)/[(optimal fiber length in cm)×(muscle density in g/cm^3^)], where muscle density is 1.06 g/cm^3^.

### Diaphragm Tetanic Contraction and Muscle Fatigue Methods

As a second approach, the diaphragm will be tested as a target for a therapeutic outcome measure. SJL-Dysf, A/J, and 129-Dysf^−/−^ animals along with control animals C57/BL10, A/HeJ, and 129S1/SvImJ (8 per group) were assessed at 24 weeks. Mice were euthanized and the diaphragm was dissected with rib attachments and central tendon intact, and placed in K-H buffer at 37 C as previously described [47], [48]. A 1–2 mm wide section (with length from rib to tendon) of diaphragm was isolated, and attached to a force transducer. The diaphragm strip was looped around a basket assembly attached to the transducer (the rib cartilage serves as the anchor), and the tendon was pierced by a pin. The muscle was stretched to optimal length for measurement of twitch contractions, and then allowed to rest for 10 minutes before initiation of the tetanic protocol. The protocol consisted of a series of eight tetanic contractions occurring at 2 minute intervals, each with duration of 500 ms. The force was recorded for each stimulus, and normalized to account for muscle width and length. The muscle was rested for 5 minutes before starting the muscle fatigue protocol. This protocol measures the force exerted by the muscle when stimulated every second for 90 seconds (500 ms tetanus at 100 Hz). Following the muscle fatigue protocol, the muscle strip was removed from the apparatus, the rib cartilage removed and weighed.

## Supporting Information

Figure S1 **RNA analysis from injected tissue.** Sequence analysis of cDNA from rAAV.DYSF-injected muscle. cDNA from injected tissues was sequenced completely and then aligned to the reference Dysferlin sequence containing UTRs. The sequenced cDNA aligns exactly with the reference.
(PDF)

Figure S2 **Functional assessment of the EDL muscle in Dysf^−^/^−^ mice.** SJL-Dysf, A/J, and 129-Dysf^−/−^ animals along with control animals C57/BL10, A/HeJ, and 129S1/SvImJ (8 per group) were assessed for physiological deficits in the EDL. (A) Dysferlin deficient muscles showed no deficits in maximum isometric force compared to strain controls when normalizing for the cross-sectional area of the muscle (ANOVA, P>0.05). (B–D) Muscles were subjected to mechanical damage by 10 repetitive eccentric contractions. Dysferlin deficient muscles were not significantly more affected (larger loss of force) by repetitive eccentric contractions compared to their corresponding strain control muscles (2-way analysis of variance, P>0.05)
(TIF)
