# Supplementary material for: Homologous Recombination Mediates Functional Recovery of Dysferlin Deficiency following AAV5 Gene Transfer
Source: PLoS One. 2012 Jun 15;7(6):e39233. doi: 10.1371/journal.pone.0039233 (PMC3376115; doi:10.1371/journal.pone.0039233)
Supplement: Figure S1 — RNA analysis from injected tissue. Sequence analysis of cDNA from rAAV.DYSF-injected muscle. cDNA from injected tissues was sequenced completely and then aligned to the reference Dysferlin sequence containing UTRs. The sequenced cDNA aligns exactly with the reference. (PDF) [file pone.0039233.s001.pdf]

Figure S1

```
Reference       GGGTGGAAGATGAGCAGAAGCCCCTGTTCTCGGAACGCCGGCTGACAAGCGGGGTGAGCG 60
Sequenced       ------------------------------------------------------------


Reference       CAGCCGGGGCGGGGACCCAGCCTAGCCCACTGGAGCAGCCGGGGGTGGCCCGTTCCCCTT 120
Sequenced       ------------------------------------------------------------


Reference       TAAGAGCAACTGCTCTAAGCCAGGAGCCAGAGATTCGAGCCGGCCTCGCCCAGCCAGCCC 180
Sequenced       ------------------------------------------------------------


Reference       TCTCCAGCGAGGGGACCCACAAGCGGCGCCTCGGCCCTCCCGACCTTTCCGAGCCCTCTT 240
Sequenced       ------------------------------------------------------------


Reference       TGCGCCCTGGGCGCACGGGGCCCTACACGCGCCAAGCATGCTGAGGGTCTTCATCCTCTA 300
Sequenced       -------------------------------------ATGCTGAGGGTCTTCATCCTCTA 23
                                                     ***********************

Reference       TGCCGAGAACGTCCACACACCCGACACCGACATCAGCGATGCCTACTGCTCCGCGGTGTT 360
Sequenced       TGCCGAGAACGTCCACACACCCGACACCGACATCAGCGATGCCTACTGCTCCGCGGTGTT 83
                ************************************************************

Reference       TGCAGGGGTGAAGAAGAGAACCAAAGTCATCAAGAACAGCGTGAACCCTGTATGGAATGA 420
Sequenced       TGCAGGGGTGAAGAAGAGAACCAAAGTCATCAAGAACAGCGTGAACCCTGTATGGAATGA 143
                ************************************************************

Reference       GGGATTTGAATGGGACCTCAAGGGCATCCCCCTGGACCAGGGCTCTGAGCTTCATGTGGT 480
Sequenced       GGGATTTGAATGGGACCTCAAGGGCATCCCCCTGGACCAGGGCTCTGAGCTTCATGTGGT 203
                ************************************************************

Reference       GGTCAAAGACCATGAGACGATGGGGAGGAACAGGTTCCTGGGGGAAGCCAAGGTCCCACT 540
Sequenced       GGTCAAAGACCATGAGACGATGGGGAGGAACAGGTTCCTGGGGGAAGCCAAGGTCCCACT 263
                ************************************************************

Reference       CCGAGAGGTCCTCGCCACCCCTAGTCTGTCCGCCAGCTTCAATGCCCCCCTGCTGGACAC 600
Sequenced       CCGAGAGGTCCTCGCCACCCCTAGTCTGTCCGCCAGCTTCAATGCCCCCCTGCTGGACAC 323
                ************************************************************

Reference       CAAGAAGCAGCCCACAGGGGCCTCGCTGGTCCTGCAGGTGTCCTACACACCGCTGCCTGG 660
Sequenced       CAAGAAGCAGCCCACAGGGGCCTCGCTGGTCCTGCAGGTGTCCTACACACCGCTGCCTGG 383
                ************************************************************

Reference       AGCTGTGCCCCTGTTCCCGCCCCCTACTCCTCTGGAGCCCTCCCCGACTCTGCCTGACCT 720
Sequenced       AGCTGTGCCCCTGTTCCCGCCCCCTACTCCTCTGGAGCCCTCCCCGACTCTGCCTGACCT 443
                ************************************************************

Reference       GGATGTAGTGGCAGACACAGGAGGAGAGGAAGACACAGAGGACCAGGGACTCACTGGAGA 780
Sequenced       GGATGTAGTGGCAGACACAGGAGGAGAGGAAGACACAGAGGACCAGGGACTCACTGGAGA 503
                ************************************************************

Reference       TGAGGCGGAGCCATTCCTGGATCAAAGCGGAGGCCCGGGGGCTCCCACCACCCCAAGGAA 840
Sequenced       TGAGGCGGAGCCATTCCTGGATCAAAGCGGAGGCCCGGGGGCTCCCACCACCCCAAGGAA 563
                ************************************************************

Reference       ACTACCTTCACGTCCTCCGCCCCACTACCCCGGGATCAAAAGAAAGCGAAGTGCGCCTAC 900
Sequenced       ACTACCTTCACGTCCTCCGCCCCACTACCCCGGGATCAAAAGAAAGCGAAGTGCGCCTAC 623
                ************************************************************

Reference       ATCTAGAAAGCTGCTGTCAGACAAACCGCAGGATTTCCAGATCAGGGTCCAGGTGATCGA 960
Sequenced       ATCTAGAAAGCTGCTGTCAGACAAACCGCAGGATTTCCAGATCAGGGTCCAGGTGATCGA 683
                ************************************************************

Reference       GGGGCGCCAGCTGCCGGGGGTGAACATCAAGCCTGTGGTCAAGGTTACCGCTGCAGGGCA 1020
Sequenced       GGGGCGCCAGCTGCCGGGGGTGAACATCAAGCCTGTGGTCAAGGTTACCGCTGCAGGGCA 743
                ************************************************************

Reference       GACCAAGCGGACGCGGATCCACAAGGGAAACAGCCCACTCTTCAATGAGACTCTTTTCTT 1080
Sequenced       GACCAAGCGGACGCGGATCCACAAGGGAAACAGCCCACTCTTCAATGAGACTCTTTTCTT 803
                ************************************************************

Reference       CAACTTGTTTGACTCTCCTGGGGAGCTGTTTGATGAGCCCATCTTTATCACGGTGGTAGA 1140
Sequenced       CAACTTGTTTGACTCTCCTGGGGAGCTGTTTGATGAGCCCATCTTTATCACGGTGGTAGA 863
                ************************************************************

Reference       CTCTCGTTCTCTCAGGACAGATGCTCTCCTCGGGGAGTTCCGGATGGACGTGGGCACCAT 1200
```

```
Sequenced      CTCTCGTTCTCTCAGGACAGATGCTCTCCTCGGGGAGTTCCGGATGGACGTGGGCACCAT 923
               ************************************************************

Reference      TTACAGAGAGCCCCGGCACGCCTATCTCAGGAAGTGGCTGCTGCTCTCAGACCCTGATGA 1260
Sequenced      TTACAGAGAGCCCCGGCACGCCTATCTCAGGAAGTGGCTGCTGCTCTCAGACCCTGATGA 983
               ************************************************************

Reference      CTTCTCTGCTGGGGCCAGAGGCTACCTGAAAACAAGCCTTTGTGTGCTGGGGCCTGGGGA 1320
Sequenced      CTTCTCTGCTGGGGCCAGAGGCTACCTGAAAACAAGCCTTTGTGTGCTGGGGCCTGGGGA 1043
               ************************************************************

Reference      CGAAGCGCCTCTGGAGAGAAAAGACCCCTCTGAAGACAAGGAGGACATTGAAAGCAACCT 1380
Sequenced      CGAAGCGCCTCTGGAGAGAAAAGACCCCTCTGAAGACAAGGAGGACATTGAAAGCAACCT 1103
               ************************************************************

Reference      GCTCCGGCCCACAGGCGTAGCCCTGCGAGGAGCCCACTTCTGCCTGAAGGTCTTCCGGGC 1440
Sequenced      GCTCCGGCCCACAGGCGTAGCCCTGCGAGGAGCCCACTTCTGCCTGAAGGTCTTCCGGGC 1163
               ************************************************************

Reference      CGAGGACTTGCCGCAGATGGACGATGCCGTGATGGACAACGTGAAACAGATCTTTGGCTT 1500
Sequenced      CGAGGACTTGCCGCAGATGGACGATGCCGTGATGGACAACGTGAAACAGATCTTTGGCTT 1223
               ************************************************************

Reference      CGAGAGTAACAAGAAGAACTTGGTGGACCCCTTTGTGGAGGTCAGCTTTGCGGGGAAAAT 1560
Sequenced      CGAGAGTAACAAGAAGAACTTGGTGGACCCCTTTGTGGAGGTCAGCTTTGCGGGGAAAAT 1283
               ************************************************************

Reference      GCTGTGCAGCAAGATCTTGGAGAAGACGGCCAACCCTCAGTGGAACCAGAACATCACACT 1620
Sequenced      GCTGTGCAGCAAGATCTTGGAGAAGACGGCCAACCCTCAGTGGAACCAGAACATCACACT 1343
               ************************************************************

Reference      GCCTGCCATGTTTCCCTCCATGTGCGAAAAAATGAGGATTCGTATCATAGACTGGGACCG 1680
Sequenced      GCCTGCCATGTTTCCCTCCATGTGCGAAAAAATGAGGATTCGTATCATAGACTGGGACCG 1403
               ************************************************************

Reference      CCTGACTCACAATGACATCGTGGCTACCACCTACCTGAGTATGTCGAAAATCTCTGCCCC 1740
Sequenced      CCTGACTCACAATGACATCGTGGCTACCACCTACCTGAGTATGTCGAAAATCTCTGCCCC 1463
               ************************************************************

Reference      TGGAGGAGAAATAGAAGAGGAGCCTGCAGGTGCTGTCAAGCCTTCGAAAGCCTCAGACTT 1800
Sequenced      TGGAGGAGAAATAGAAGAGGAGCCTGCAGGTGCTGTCAAGCCTTCGAAAGCCTCAGACTT 1523
               ************************************************************

Reference      GGATGACTACCTGGGCTTCCTCCCCACTTTTGGGCCCTGCTACATCAACCTCTATGGCAG 1860
Sequenced      GGATGACTACCTGGGCTTCCTCCCCACTTTTGGGCCCTGCTACATCAACCTCTATGGCAG 1583
               ************************************************************

Reference      TCCCAGAGAGTTCACAGGCTTCCCAGACCCCTACACAGAGCTCAACACAGGCAAGGGGGA 1920
Sequenced      TCCCAGAGAGTTCACAGGCTTCCCAGACCCCTACACAGAGCTCAACACAGGCAAGGGGGA 1643
               ************************************************************

Reference      AGGTGTGGCTTATCGTGGCCGGCTTCTGCTCTCCCTGGAGACCAAGCTGGTGGAGCACAG 1980
Sequenced      AGGTGTGGCTTATCGTGGCCGGCTTCTGCTCTCCCTGGAGACCAAGCTGGTGGAGCACAG 1703
               ************************************************************

Reference      TGAACAGAAGGTGGAGGACCTTCCTGCGGATGACATCCTCCGGGTGGAGAAGTACCTTAG 2040
Sequenced      TGAACAGAAGGTGGAGGACCTTCCTGCGGATGACATCCTCCGGGTGGAGAAGTACCTTAG 1763
               ************************************************************

Reference      GAGGCGCAAGTACTCCCTGTTTGCGGCCTTCTACTCAGCCACCATGCTGCAGGATGTGGA 2100
Sequenced      GAGGCGCAAGTACTCCCTGTTTGCGGCCTTCTACTCAGCCACCATGCTGCAGGATGTGGA 1823
               ************************************************************

Reference      TGATGCCATCCAGTTTGAGGTCAGCATCGGGAACTACGGGAACAAGTTCGACATGACCTG 2160
Sequenced      TGATGCCATCCAGTTTGAGGTCAGCATCGGGAACTACGGGAACAAGTTCGACATGACCTG 1883
               ************************************************************

Reference      CCTGCCGCTGGCCTCCACCACTCAGTACAGCCGTGCAGTCTTTGACGGGTGCCACTACTA 2220
Sequenced      CCTGCCGCTGGCCTCCACCACTCAGTACAGCCGTGCAGTCTTTGACGGGTGCCACTACTA 1943
               ************************************************************

Reference      CTACCTACCCTGGGGTAACGTGAAACCTGTGGTGGTGCTGTCATCCTACTGGGAGGACAT 2280
Sequenced      CTACCTACCCTGGGGTAACGTGAAACCTGTGGTGGTGCTGTCATCCTACTGGGAGGACAT 2003
               ************************************************************

Reference      CAGCCATAGAATCGAGACTCAGAACCAGCTGCTTGGGATTGCTGACCGGCTGGAAGCTGG 2340
Sequenced      CAGCCATAGAATCGAGACTCAGAACCAGCTGCTTGGGATTGCTGACCGGCTGGAAGCTGG 2063
               ************************************************************

Reference      CCTGGAGCAGGTCCACCTGGCCCTGAAGGCGCAGTGCTCCACGGAGGACGTGGACTCGCT 2400
Sequenced      CCTGGAGCAGGTCCACCTGGCCCTGAAGGCGCAGTGCTCCACGGAGGACGTGGACTCGCT 2123
```

```
                ************************************************************

Reference       GGTGGCTCAGCTGACGGATGAGCTCATCGCAGGCTGCAGCCAGCCTCTGGGTGACATCCA 2460
Sequenced       GGTGGCTCAGCTGACGGATGAGCTCATCGCAGGCTGCAGCCAGCCTCTGGGTGACATCCA 2183
                ************************************************************

Reference       TGAGACACCCTCTGCCACCCACCTGGACCAGTACCTGTACCAGCTGCGCACCCATCACCT 2520
Sequenced       TGAGACACCCTCTGCCACCCACCTGGACCAGTACCTGTACCAGCTGCGCACCCATCACCT 2243
                ************************************************************

Reference       GAGCCAAATCACTGAGGCTGCCCTGGCCCTGAAGCTCGGCCACAGTGAGCTCCCTGCAGC 2580
Sequenced       GAGCCAAATCACTGAGGCTGCCCTGGCCCTGAAGCTCGGCCACAGTGAGCTCCCTGCAGC 2303
                ************************************************************

Reference       TCTGGAGCAGGCGGAGGACTGGCTCCTGCGTCTGCGTGCCCTGGCAGAGGAGCCCCAGAA 2640
Sequenced       TCTGGAGCAGGCGGAGGACTGGCTCCTGCGTCTGCGTGCCCTGGCAGAGGAGCCCCAGAA 2363
                ************************************************************

Reference       CAGCCTGCCGGACATCGTCATCTGGATGCTGCAGGGAGACAAGCGTGTGGCATACCAGCG 2700
Sequenced       CAGCCTGCCGGACATCGTCATCTGGATGCTGCAGGGAGACAAGCGTGTGGCATACCAGCG 2423
                ************************************************************

Reference       GGTGCCCGCCCACCAAGTCCTCTTCTCCCGGCGGGGTGCCAACTACTGTGGCAAGAATTG 2760
Sequenced       GGTGCCCGCCCACCAAGTCCTCTTCTCCCGGCGGGGTGCCAACTACTGTGGCAAGAATTG 2483
                ************************************************************

Reference       TGGGAAGCTACAGACAATCTTTCTGAAATATCCGATGGAGAAGGTGCCTGGCGCCCGGAT 2820
Sequenced       TGGGAAGCTACAGACAATCTTTCTGAAATATCCGATGGAGAAGGTGCCTGGCGCCCGGAT 2543
                ************************************************************

Reference       GCCAGTGCAGATACGGGTCAAGCTGTGGTTTGGGCTCTCTGTGGATGAGAAGGAGTTCAA 2880
Sequenced       GCCAGTGCAGATACGGGTCAAGCTGTGGTTTGGGCTCTCTGTGGATGAGAAGGAGTTCAA 2603
                ************************************************************

Reference       CCAGTTTGCTGAGGGGAAGCTGTCTGTCTTTGCTGAAACCTATGAGAACGAGACTAAGTT 2940
Sequenced       CCAGTTTGCTGAGGGGAAGCTGTCTGTCTTTGCTGAAACCTATGAGAACGAGACTAAGTT 2663
                ************************************************************

Reference       GGCCCTTGTTGGGAACTGGGGCACAACGGGCCTCACCTACCCCAAGTTTTCTGACGTCAC 3000
Sequenced       GGCCCTTGTTGGGAACTGGGGCACAACGGGCCTCACCTACCCCAAGTTTTCTGACGTCAC 2723
                ************************************************************

Reference       GGGCAAGATCAAGCTACCCAAGGACAGCTTCCGCCCCTCGGCCGGCTGGACCTGGGCTGG 3060
Sequenced       GGGCAAGATCAAGCTACCCAAGGACAGCTTCCGCCCCTCGGCCGGCTGGACCTGGGCTGG 2783
                ************************************************************

Reference       AGATTGGTTCGTGTGTCCGGAGAAGACTCTGCTCCATGACATGGACGCCGGTCACCTGAG 3120
Sequenced       AGATTGGTTCGTGTGTCCGGAGAAGACTCTGCTCCATGACATGGACGCCGGTCACCTGAG 2843
                ************************************************************

Reference       CTTCGTGGAAGAGGTGTTTGAGAACCAGACCCGGCTTCCCGGAGGCCAGTGGATCTACAT 3180
Sequenced       CTTCGTGGAAGAGGTGTTTGAGAACCAGACCCGGCTTCCCGGAGGCCAGTGGATCTACAT 2903
                ************************************************************

Reference       GAGTGACAACTACACCGATGTGAACGGGGAGAAGGTGCTTCCCAAGGATGACATTGAGTG 3240
Sequenced       GAGTGACAACTACACCGATGTGAACGGGGAGAAGGTGCTTCCCAAGGATGACATTGAGTG 2963
                ************************************************************

Reference       CCCACTGGGCTGGAAGTGGGAAGATGAGGAATGGTCCACAGACCTCAACCGGGCTGTCGA 3300
Sequenced       CCCACTGGGCTGGAAGTGGGAAGATGAGGAATGGTCCACAGACCTCAACCGGGCTGTCGA 3023
                ************************************************************

Reference       TGAGCAAGGCTGGGAGTATAGCATCACCATCCCCCCGGAGCGGAAGCCGAAGCACTGGGT 3360
Sequenced       TGAGCAAGGCTGGGAGTATAGCATCACCATCCCCCCGGAGCGGAAGCCGAAGCACTGGGT 3083
                ************************************************************

Reference       CCCTGCTGAGAAGATGTACTACACACACCGACGGCGGCGCTGGGTGCGCCTGCGCAGGAG 3420
Sequenced       CCCTGCTGAGAAGATGTACTACACACACCGACGGCGGCGCTGGGTGCGCCTGCGCAGGAG 3143
                ************************************************************

Reference       GGATCTCAGCCAAATGGAAGCACTGAAAAGGCACAGGCAGGCGGAGGCGGAGGGCGAGGG 3480
Sequenced       GGATCTCAGCCAAATGGAAGCACTGAAAAGGCACAGGCAGGCGGAGGCGGAGGGCGAGGG 3203
                ************************************************************

Reference       CTGGGAGTACGCCTCTCTTTTTGGCTGGAAGTTCCACCTCGAGTACCGCAAGACAGATGC 3540
Sequenced       CTGGGAGTACGCCTCTCTTTTTGGCTGGAAGTTCCACCTCGAGTACCGCAAGACAGATGC 3263
                ************************************************************

Reference       CTTCCGCCGCCGCCGCTGGCGCCGTCGCATGGAGCCACTGGAGAAGACGGGGCCTGCAGC 3600
Sequenced       CTTCCGCCGCCGCCGCTGGCGCCGTCGCATGGAGCCACTGGAGAAGACGGGGCCTGCAGC 3323
                ************************************************************
```

```
Reference      TGTGTTTGCCCTTGAGGGGGCCCTGGGCGGCGTGATGGATGACAAGAGTGAAGATTCCAT 3660
Sequenced      TGTGTTTGCCCTTGAGGGGGCCCTGGGCGGCGTGATGGATGACAAGAGTGAAGATTCCAT 3383
               ************************************************************

Reference      GTCCGTCTCCACCTTGAGCTTCGGTGTGAACAGACCCACGATTTCCTGCATATTCGACTA 3720
Sequenced      GTCCGTCTCCACCTTGAGCTTCGGTGTGAACAGACCCACGATTTCCTGCATATTCGACTA 3443
               ************************************************************

Reference      TGGGAACCGCTACCATCTACGCTGCTACATGTACCAGGCCCGGGACCTGGCTGCGATGGA 3780
Sequenced      TGGGAACCGCTACCATCTACGCTGCTACATGTACCAGGCCCGGGACCTGGCTGCGATGGA 3503
               ************************************************************

Reference      CAAGGACTCTTTTTCTGATCCCTATGCCATCGTCTCCTTCCTGCACCAGAGCCAGAAGAC 3840
Sequenced      CAAGGACTCTTTTTCTGATCCCTATGCCATCGTCTCCTTCCTGCACCAGAGCCAGAAGAC 3563
               ************************************************************

Reference      GGTGGTGGTGAAGAACACCCTTAACCCCACCTGGGACCAGACGCTCATCTTCTACGAGAT 3900
Sequenced      GGTGGTGGTGAAGAACACCCTTAACCCCACCTGGGACCAGACGCTCATCTTCTACGAGAT 3623
               ************************************************************

Reference      CGAGATCTTTGGCGAGCCGGCCACAGTTGCTGAGCAACCGCCCAGCATTGTGGTGGAGCT 3960
Sequenced      CGAGATCTTTGGCGAGCCGGCCACAGTTGCTGAGCAACCGCCCAGCATTGTGGTGGAGCT 3683
               ************************************************************

Reference      GTACGACCATGACACTTATGGTGCAGACGAGTTTATGGGTCGCTGCATCTGTCAACCGAG 4020
Sequenced      GTACGACCATGACACTTATGGTGCAGACGAGTTTATGGGTCGCTGCATCTGTCAACCGAG 3743
               ************************************************************

Reference      TCTGGAACGGATGCCACGGCTGGCCTGGTTCCCACTGACGAGGGGCAGCCAGCCGTCGGG 4080
Sequenced      TCTGGAACGGATGCCACGGCTGGCCTGGTTCCCACTGACGAGGGGCAGCCAGCCGTCGGG 3803
               ************************************************************

Reference      GGAGCTGCTGGCCTCTTTTGAGCTCATCCAGAGAGAGAAGCCGGCCATCCACCATATTCC 4140
Sequenced      GGAGCTGCTGGCCTCTTTTGAGCTCATCCAGAGAGAGAAGCCGGCCATCCACCATATTCC 3863
               ************************************************************

Reference      TGGTTTTGAGGTGCAGGAGACATCAAGGATCCTGGATGAGTCTGAGGACACAGACCTGCC 4200
Sequenced      TGGTTTTGAGGTGCAGGAGACATCAAGGATCCTGGATGAGTCTGAGGACACAGACCTGCC 3923
               ************************************************************

Reference      CTACCCACCACCCCAGAGGGAGGCCAACATCTACATGGTTCCTCAGAACATCAAGCCAGC 4260
Sequenced      CTACCCACCACCCCAGAGGGAGGCCAACATCTACATGGTTCCTCAGAACATCAAGCCAGC 3983
               ************************************************************

Reference      GCTCCAGCGTACCGCCATCGAGATCCTGGCATGGGGCCTGCGGAACATGAAGAGTTACCA 4320
Sequenced      GCTCCAGCGTACCGCCATCGAGATCCTGGCATGGGGCCTGCGGAACATGAAGAGTTACCA 4043
               ************************************************************

Reference      GCTGGCCAACATCTCCTCCCCCAGCCTCGTGGTAGAGTGTGGGGGCCAGACGGTGCAGTC 4380
Sequenced      GCTGGCCAACATCTCCTCCCCCAGCCTCGTGGTAGAGTGTGGGGGCCAGACGGTGCAGTC 4103
               ************************************************************

Reference      CTGTGTCATCAGGAACCTCCGGAAGAACCCCAACTTTGACATCTGCACCCTCTTCATGGA 4440
Sequenced      CTGTGTCATCAGGAACCTCCGGAAGAACCCCAACTTTGACATCTGCACCCTCTTCATGGA 4163
               ************************************************************

Reference      AGTGATGCTGCCCAGGGAGGAGCTCTACTGCCCCCCCATCACCGTCAAGGTCATCGATAA 4500
Sequenced      AGTGATGCTGCCCAGGGAGGAGCTCTACTGCCCCCCCATCACCGTCAAGGTCATCGATAA 4223
               ************************************************************

Reference      CCGCCAGTTTGGCCGCCGGCCTGTGGTGGGCCAGTGTACCATCCGCTCCCTGGAGAGCTT 4560
Sequenced      CCGCCAGTTTGGCCGCCGGCCTGTGGTGGGCCAGTGTACCATCCGCTCCCTGGAGAGCTT 4283
               ************************************************************

Reference      CCTGTGTGACCCCTACTCGGCGGAGAGTCCATCCCCACAGGGTGGCCCAGACGATGTGAG 4620
Sequenced      CCTGTGTGACCCCTACTCGGCGGAGAGTCCATCCCCACAGGGTGGCCCAGACGATGTGAG 4343
               ************************************************************

Reference      CCTACTCAGTCCTGGGGAAGACGTGCTCATCGACATTGATGACAAGGAGCCCCTCATCCC 4680
Sequenced      CCTACTCAGTCCTGGGGAAGACGTGCTCATCGACATTGATGACAAGGAGCCCCTCATCCC 4403
               ************************************************************

Reference      CATCCAGGAGGAAGAGTTCATCGATTGGTGGAGCAAATTCTTTGCCTCCATAGGGGAGAG 4740
Sequenced      CATCCAGGAGGAAGAGTTCATCGATTGGTGGAGCAAATTCTTTGCCTCCATAGGGGAGAG 4463
               ************************************************************

Reference      GGAAAAGTGCGGCTCCTACCTGGAGAAGGATTTTGACACCCTGAAGGTCTATGACACACA 4800
Sequenced      GGAAAAGTGCGGCTCCTACCTGGAGAAGGATTTTGACACCCTGAAGGTCTATGACACACA 4523
               ************************************************************
```

```
Reference      GCTGGAGAATGTGGAGGCCTTTGAGGGCCTGTCTGACTTTTGTAACACCTTCAAGCTGTA 4860
Sequenced      GCTGGAGAATGTGGAGGCCTTTGAGGGCCTGTCTGACTTTTGTAACACCTTCAAGCTGTA 4583
               ************************************************************

Reference      CCGGGGCAAGACGCAGGAGGAGACAGAAGATCCATCTGTGATTGGTGAATTTAAGGGCCT 4920
Sequenced      CCGGGGCAAGACGCAGGAGGAGACAGAAGATCCATCTGTGATTGGTGAATTTAAGGGCCT 4643
               ************************************************************

Reference      CTTCAAAATTTATCCCCTCCCAGAAGACCCAGCCATCCCCATGCCCCCAAGACAGTTCCA 4980
Sequenced      CTTCAAAATTTATCCCCTCCCAGAAGACCCAGCCATCCCCATGCCCCCAAGACAGTTCCA 4703
               ************************************************************

Reference      CCAGCTGGCCGCCCAGGGACCCCAGGAGTGCTTGGTCCGTATCTACATTGTCCGAGCATT 5040
Sequenced      CCAGCTGGCCGCCCAGGGACCCCAGGAGTGCTTGGTCCGTATCTACATTGTCCGAGCATT 4763
               ************************************************************

Reference      TGGCCTGCAGCCCAAGGACCCCAATGGAAAGTGTGATCCTTACATCAAGATCTCCATAGG 5100
Sequenced      TGGCCTGCAGCCCAAGGACCCCAATGGAAAGTGTGATCCTTACATCAAGATCTCCATAGG 4823
               ************************************************************

Reference      GAAGAAATCAGTGAGTGACCAGGATAACTACATCCCCTGCACGCTGGAGCCCGTATTTGG 5160
Sequenced      GAAGAAATCAGTGAGTGACCAGGATAACTACATCCCCTGCACGCTGGAGCCCGTATTTGG 4883
               ************************************************************

Reference      AAAGATGTTCGAGCTGACCTGCACTCTGCCTCTGGAGAAGGACCTAAAGATCACTCTCTA 5220
Sequenced      AAAGATGTTCGAGCTGACCTGCACTCTGCCTCTGGAGAAGGACCTAAAGATCACTCTCTA 4943
               ************************************************************

Reference      TGACTATGACCTCCTCTCCAAGGACGAAAAGATCGGTGAGACGGTCGTCGACCTGGAGAA 5280
Sequenced      TGACTATGACCTCCTCTCCAAGGACGAAAAGATCGGTGAGACGGTCGTCGACCTGGAGAA 5003
               ************************************************************

Reference      CAGGCTGCTGTCCAAGTTTGGGGCTCGCTGTGGACTCCCACAGACCTACTGTGTCTCTGG 5340
Sequenced      CAGGCTGCTGTCCAAGTTTGGGGCTCGCTGTGGACTCCCACAGACCTACTGTGTCTCTGG 5063
               ************************************************************

Reference      ACCGAACCAGTGGCGGGACCAGCTCCGCCCCTCCCAGCTCCTCCACCTCTTCTGCCAGCA 5400
Sequenced      ACCGAACCAGTGGCGGGACCAGCTCCGCCCCTCCCAGCTCCTCCACCTCTTCTGCCAGCA 5123
               ************************************************************

Reference      GCATAGAGTCAAGGCACCTGTGTACCGGACAGACCGTGTAATGTTTCAGGATAAAGAATA 5460
Sequenced      GCATAGAGTCAAGGCACCTGTGTACCGGACAGACCGTGTAATGTTTCAGGATAAAGAATA 5183
               ************************************************************

Reference      TTCCATTGAAGAGATAGAGGCTGGCAGGATCCCAAACCCACACCTGGGCCCAGTGGAGGA 5520
Sequenced      TTCCATTGAAGAGATAGAGGCTGGCAGGATCCCAAACCCACACCTGGGCCCAGTGGAGGA 5243
               ************************************************************

Reference      GCGTCTGGCTCTGCATGTGCTTCAGCAGCAGGGCCTGGTCCCGGAGCACGTGGAGTCACG 5580
Sequenced      GCGTCTGGCTCTGCATGTGCTTCAGCAGCAGGGCCTGGTCCCGGAGCACGTGGAGTCACG 5303
               ************************************************************

Reference      GCCCCTCTACAGCCCCCTGCAGCCAGACATCGAGCAGGGGAAGCTGCAGATGTGGGTCGA 5640
Sequenced      GCCCCTCTACAGCCCCCTGCAGCCAGACATCGAGCAGGGGAAGCTGCAGATGTGGGTCGA 5363
               ************************************************************

Reference      CCTATTTCCGAAGGCCCTGGGGCGGCCTGGACCTCCCTTCAACATCACCCCACGGAGAGC 5700
Sequenced      CCTATTTCCGAAGGCCCTGGGGCGGCCTGGACCTCCCTTCAACATCACCCCACGGAGAGC 5423
               ************************************************************

Reference      CAGAAGGTTTTTCCTGCGTTGTATTATCTGGAATACCAGAGATGTGATCCTGGATGACCT 5760
Sequenced      CAGAAGGTTTTTCCTGCGTTGTATTATCTGGAATACCAGAGATGTGATCCTGGATGACCT 5483
               ************************************************************

Reference      GAGCCTCACGGGGGAGAAGATGAGCGACATTTATGTGAAAGGTTGGATGATTGGCTTTGA 5820
Sequenced      GAGCCTCACGGGGGAGAAGATGAGCGACATTTATGTGAAAGGTTGGATGATTGGCTTTGA 5543
               ************************************************************

Reference      AGAACACAAGCAAAAGACAGACGTGCATTATCGTTCCCTGGGAGGTGAAGGCAACTTCAA 5880
Sequenced      AGAACACAAGCAAAAGACAGACGTGCATTATCGTTCCCTGGGAGGTGAAGGCAACTTCAA 5603
               ************************************************************

Reference      CTGGAGGTTCATTTTCCCCTTCGACTACCTGCCAGCTGAGCAAGTCTGTACCATTGCCAA 5940
Sequenced      CTGGAGGTTCATTTTCCCCTTCGACTACCTGCCAGCTGAGCAAGTCTGTACCATTGCCAA 5663
               ************************************************************

Reference      GAAGGATGCCTTCTGGAGGCTGGACAAGACTGAGAGCAAAATCCCAGCACGAGTGGTGTT 6000
Sequenced      GAAGGATGCCTTCTGGAGGCTGGACAAGACTGAGAGCAAAATCCCAGCACGAGTGGTGTT 5723
               ************************************************************

Reference      CCAGATCTGGGACAATGACAAGTTCTCCTTTGATGATTTTCTGGGCTCCCTGCAGCTCGA 6060
```

```
Sequenced       CCAGATCTGGGACAATGACAAGTTCTCCTTTGATGATTTTCTGGGCTCCCTGCAGCTCGA 5783
                ************************************************************

Reference       TCTCAACCGCATGCCCAAGCCAGCCAAGACAGCCAAGAAGTGCTCCTTGGACCAGCTGGA 6120
Sequenced       TCTCAACCGCATGCCCAAGCCAGCCAAGACAGCCAAGAAGTGCTCCTTGGACCAGCTGGA 5843
                ************************************************************

Reference       TGATGCTTTCCACCCAGAATGGTTTGTGTCCCTTTTTGAGCAGAAAACAGTGAAGGGCTG 6180
Sequenced       TGATGCTTTCCACCCAGAATGGTTTGTGTCCCTTTTTGAGCAGAAAACAGTGAAGGGCTG 5903
                ************************************************************

Reference       GTGGCCCTGTGTAGCAGAAGAGGGTGAGAAGAAAATACTGGCGGGCAAGCTGGAAATGAC 6240
Sequenced       GTGGCCCTGTGTAGCAGAAGAGGGTGAGAAGAAAATACTGGCGGGCAAGCTGGAAATGAC 5963
                ************************************************************

Reference       CTTGGAGATTGTAGCAGAGAGTGAGCATGAGGAGCGGCCTGCTGGCCAGGGCCGGGATGA 6300
Sequenced       CTTGGAGATTGTAGCAGAGAGTGAGCATGAGGAGCGGCCTGCTGGCCAGGGCCGGGATGA 6023
                ************************************************************

Reference       GCCCAACATGAACCCTAAGCTTGAGGACCCAAGGCGCCCCGACACCTCCTTCCTGTGGTT 6360
Sequenced       GCCCAACATGAACCCTAAGCTTGAGGACCCAAGGCGCCCCGACACCTCCTTCCTGTGGTT 6083
                ************************************************************

Reference       TACCTCCCCATACAAGACCATGAAGTTCATCCTGTGGCGGCGTTTCCGGTGGGCCATCAT 6420
Sequenced       TACCTCCCCATACAAGACCATGAAGTTCATCCTGTGGCGGCGTTTCCGGTGGGCCATCAT 6143
                ************************************************************

Reference       CCTCTTCATCATCCTCTTCATCCTGCTGCTGTTCCTGGCCATCTTCATCTACGCCTTCCC 6480
Sequenced       CCTCTTCATCATCCTCTTCATCCTGCTGCTGTTCCTGGCCATCTTCATCTACGCCTTCCC 6203
                ************************************************************

Reference       GAACTATGCTGCCATGAAGCTGGTGAAGCCCTTCAGCTGAGGACTCTCCTGCCCTGTAGA 6540
Sequenced       GAACTATGCTGCCATGAAGCTGGTGAAGCCCTTCAGCTGA-------------------- 6243
                ****************************************

Reference       AGGGGCCGTGGGGTCCCCTCCAGCATGGGACTGGCCTGCCTCCTCCGCCCAGCTCGGCGA 6600
Sequenced       ------------------------------------------------------------

Reference       GCTCCTCCAGACCTCCTAGGCCTGATTGTCCTGCCAGGGTGGGCAGACAGACAGATGGAC 6660
Sequenced       ------------------------------------------------------------

Reference       CGGCCCACACTCCCAGAGTTGCTAACATGGAGCTCTGAGATCACCCCACTTCCATCATTT 6720
Sequenced       ------------------------------------------------------------

Reference       CCTTCTCCCCCAACCCAACGCTTTTTTGGATCAGCTCAGACATATTTCAGTATAAAACAG 6780
Sequenced       ------------------------------------------------------------

Reference       TTGGAACCACACAGCA 6796
Sequenced       ----------------
```
