# Supplementary figures and images for: Homologous Recombination Mediates Functional Recovery of Dysferlin Deficiency following AAV5 Gene Transfer
Source: PLoS One. 2012 Jun 15;7(6):e39233. doi: 10.1371/journal.pone.0039233 (PMC3376115; doi:10.1371/journal.pone.0039233)

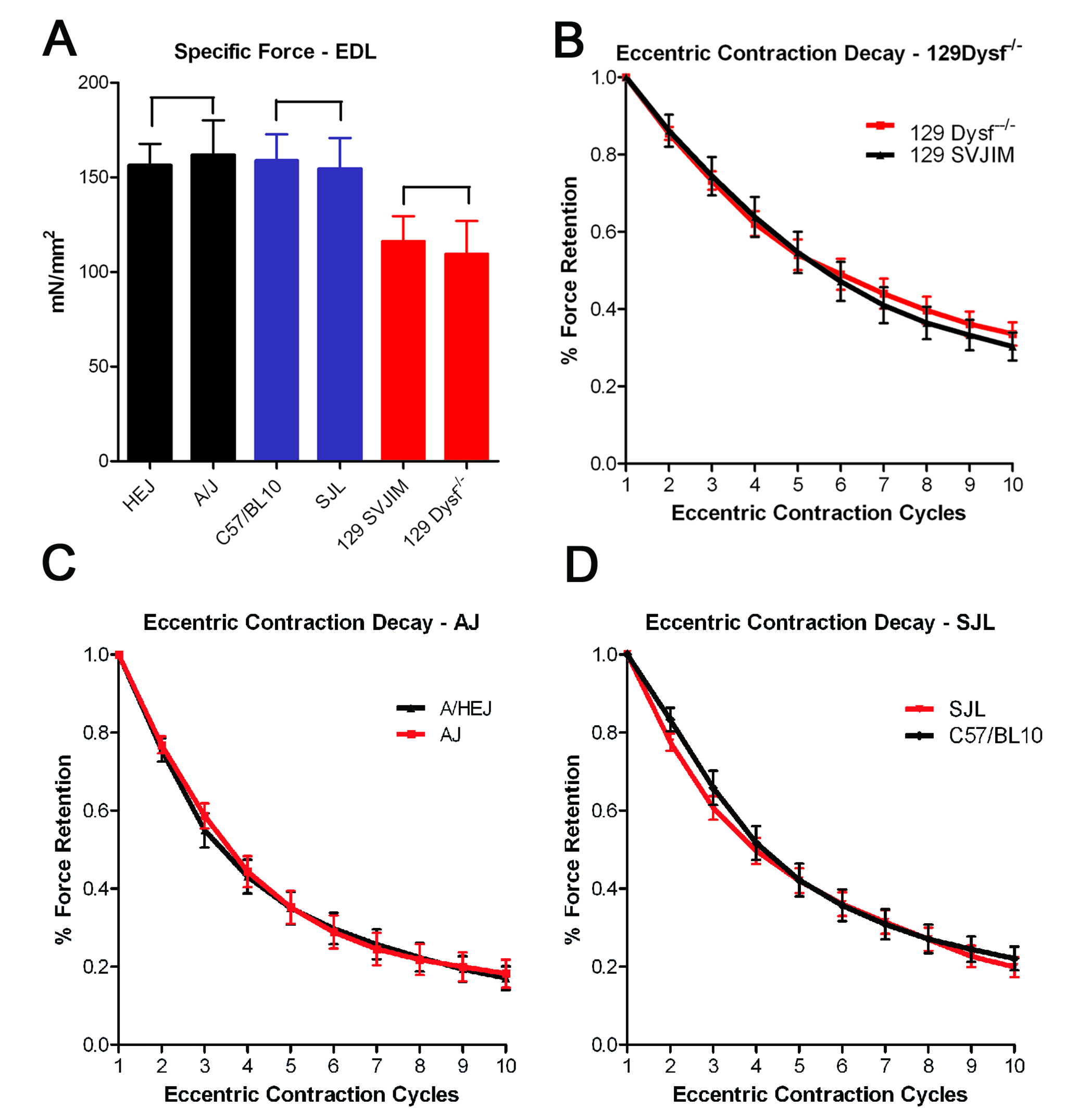

Supplement: Figure S2 — Functional assessment of the EDL muscle in Dysf−/− mice. SJL-Dysf, A/J, and 129-Dysf−/− animals along with control animals C57/BL10, A/HeJ, and 129S1/SvImJ (8 per group) were assessed for physiological deficits in the EDL. (A) Dysferlin deficient muscles showed no deficits in maximum isometric force compared to strain controls when normalizing for the cross-sectional area of the muscle (ANOVA, P>0.05). (B–D) Muscles were subjected to mechanical damage by 10 repetitive eccentric contractions. Dysferlin deficient muscles were not significantly more affected (larger loss of force) by repetitive eccentric contractions compared to their corresponding strain control muscles (2-way analysis of variance, P>0.05) (TIF) [file pone.0039233.s002.tif]
